# Cross-sectional evaluation of patients with refractory or unexplained chronic cough by office-based pulmonologists in Germany

**DOI:** 10.3389/fmed.2025.1623378

**Published:** 2025-08-04

**Authors:** Peter Kardos, Heinrich Worth, Ulrich Koehler, Martin Horack, Barbara Karmann, Christina Jannowitz, Adrian Gillissen

**Affiliations:** ^1^Lungenzentrum Frankfurt, Frankfurt am Main, Germany; ^2^Pneumologische and Kardiologische Praxisgemeinschaft, Fürth, Germany; ^3^Schlafmedizinisches Zentrum, Klinik für Pneumologie, Universitätsklinikum Marburg, Marburg, Germany; ^4^Institut für Herzinfarktforschung, Ludwigshafen am Rhein, Germany; ^5^MSD Sharp & Dohme GmbH, Medical Department, München, Germany; ^6^Department of Pulmonary Medicine and Intensive Care, Klinikum Stuttgart – Katharinenhospital, Stuttgart, Germany

**Keywords:** chronic cough, prevalence of subtypes, causes, actual management, observational study

## Abstract

**Background:**

Chronic cough (CC) is common in clinical practice, with refractory chronic cough (RCC) and unexplained chronic cough (UCC) being defined by guidelines from the European Respiratory Society, German Respiratory Society (DGP), and German Society of General Practice and Family Medicine (DEGAM) as separate entities. This study aimed to investigate the prevalence of RCC/UCC in an outpatient setting of pulmonologists.

**Methods:**

This cross-sectional observational study was conducted in 16 pulmonologists’ offices in Germany. Adult patients with a specialist’s diagnosis of RCC or UCC and who provided informed consent were included. Active smokers were excluded.

**Results:**

Of 22,140 consecutive out-patients screened, 421 were eligible for general analysis, and 226 met the RCC/UCC criteria per DGP guidelines for prevalence analysis. Among the 421 patients, 71.3% were female, 77.9% had therapy-resistant chronic cough (TRCC), and 22.1% had UCC. The main causes of TRCC were otherwise controlled asthma (59.1%) and gastroesophageal reflux disease (19.1%). Diagnosis had been established on average 6.4 ± 7.6 years prior. Common medications included inhaled corticosteroids (ICS/LABA: 45.6%, ICS mono: 26.8%), and herbal antitussives (23.3%). Codeine was used in 4.3% and morphine in 0.7%. Non-drug therapies like physiotherapy were infrequently used. Prevalence projections for RCC/UCC in Germany were 0.21 and 0.64%, based on different models.

**Conclusion:**

This study, the first in a secondary care setting in Germany to determine RCC/UCC prevalence in outpatients, found a higher prevalence in women and a long history of suffering. These findings underscore the need for improved diagnostic procedures and new therapeutic developments for RCC/UCC. Key limitations include the potential impact of the Covid-19 pandemic on data collection, a relatively small UCC sample, and potential referral bias due to the secondary care setting.

## Background

Cough is one of the most common symptoms prompting patients to seek advice from primary care physicians worldwide (1, 2). The global significance of cough as a clinical issue has led to the publication of numerous clinical guidelines by international and national societies to aid in its diagnosis and management (3–5). These guidelines categorize cough based on its duration: acute (<3 weeks), sub-acute (3–8 weeks), and chronic (>8 weeks), with specific diagnostic options recommended for each category. Acute cough is typically associated with acute viral upper respiratory tract infections, while sub-acute and chronic coughs have different underlying etiologies (3, 5, 6).

Terminology for refractory chronic cough (RCC) is not always clear-cut, as various overlapping terms such as chronic cough (CC), unexplained chronic cough (UCC), and RCC have been used to describe the same clinical condition (7). RCC is defined as a cough refractory to conventional treatments targeting potential underlying causes. The German Respiratory Society (DGP) guidelines distinguish RCC from chronic idiopathic cough (UCC), with RCC persisting despite targeted treatment of underlying diseases and UCC having an unknown etiology.

A decade ago, the concept of CC due to cough reflex hypersensitivity was introduced (8). However, reliable data on this phenomenon are still scarce, highlighting the need for further research and better understanding of underlying mechanisms of CC.

Depending on the country and health care setting, CC has been estimated to affect 4–12% of the general adult population (9, 10). In Germany, a recent cross-sectional study found a 12-month prevalence of 4.9% (11). Very little is known of the natural history of CC (3). For approximately two-thirds of affected patients, a chest X-ray and a lung function test identify the underlying co-morbid condition of the symptom cough which then can be effectively managed by optimizing the therapy for the underlying condition (12).

However, a minority of patients with potential underlying co-morbid conditions cannot be effectively managed despite adequate therapy for those conditions and are considered to have RCC, which can result from one or more underlying medical conditions such as asthma (cough as an asthma equivalent without bronchial obstruction), non-asthmatic eosinophilic bronchitis, upper airway cough syndrome (including rhinosinusitis and pharyngolaryngitis), and gastro-oesophageal reflux (5). Additionally, patients with otherwise controlled asthma, as per GINA guidelines, may still experience treatment-resistant cough (13).

In addition, in 5–10% of patients, the cause of the CC remains completely unexplained despite thorough diagnostic evaluation, classifying these cases as UCC, sometimes also termed idiopathic chronic cough (7, 14, 15). The prevalence of possible RCC/UCC has been estimated at 1% of the total Swedish population (16), and at 3.3% in a recent Canadian study (17).

At the time of this study no medication was available in Europe and the US for treatment of RCC and UCC. Synthetic and some herbal antitussives which have limited efficacy in CC were only approved for short-term use in acute and subacute cough. Given the prolonged nature, associated morbidity, and lack of effective treatments, RCC and UCC represent significant unmet needs.

Data from patients with RCC or UCC in clinical practice are not yet readily available because it was only in January 2022 that these conditions were assigned an ICD code in the German version of ICD-10 (R05 and U.69.6). Efforts are ongoing to include an ICD code in the ICD-11 revision.

In Germany, three recent cough guidelines are in use: the German Respiratory Society (DGP) 2019 guideline (5), which details the diagnostic pathway for pulmonologists, the guideline by the German Society of General Medicine and Family Medicine (DEGAM) 2021 guideline (6) and the ERS guideline (3). There are only 934 office-based pulmonologists in Germany, which limits accessibility for patients with respiratory problems to this expert group (14, 15). Furthermore, at the time of this study, there was limited awareness of RCC and UCC as distinct entities and insufficient implementation of the cough guidelines for these conditions. The prolonged nature, associated morbidity, and lack of effective treatments underscore the significant unmet medical need for patients suffering from RCC and UCC.

Against this background, we aimed to collect detailed data on RCC and UCC patients in an outpatient setting managed by pulmonologists in Germany. Our objectives included gathering information on patient characteristics, co-morbidities, diagnostic work-up, ICD-10 codes used for documentation, and treatment choices. Furthermore, we aimed to extrapolate these data to estimate the prevalence of the conditions in the adult population in Germany.

## Methods

### Ethical and legal aspects

The VICHAS (“Evaluation of chronic cough patients in a routine setting in clinical practice to identify the prevalence and incidence in Germany”) study protocol was first approved by the institutional review board of the Bavarian Board of Physicians in Munich on 27 August 2019 (No. 19051), and consequently by all other ethic committees with participating sites. All patients provided written informed consent. The study was conducted in accordance with the ethical principles of the Declaration of Helsinki. The patients’ privacy was kept according to the requirements of Directive 95/46 EC and national legislation for data protection. Data were collected in a pseudonymous way. Responsible party (legal sponsor) was MSD Sharp & Dohme GmbH, Germany.

### Design and setting

VICHAS was a non-interventional, prospective, cross-sectional multicentre study conducted from 01.01. – 31.12.2020. Consecutive patients presenting with CC in 16 pulmonologists’ offices across Germany were documented. The study was designed as an epidemiological data collection without any intervention. Selection of study sites was based on criteria to achieve balance in terms of geographic location, number of eligible patients and availability of target patient group.

All patients visiting the selected physician’s office were asked to fill in a standardized data collection form for the assessment of cough, lasting >8 weeks, independent of the possible etiology. According to the form the physicians classified them into “no cough,” “CC,” and specific subgroups “RCC” or “UCC.”

Despite the DGP guidelines for cough have been already published 2019, the collected data from 2020 showed, yet the RCC/UCC categorization was not implemented in secondary care. The group categorized as RCC patients included diagnoses which were not suitable for the guideline definition of this subgroup (e.g., ILD, COPD). For the prevalence estimation we had to define a group of RCC and UCC patients who fulfill the DGP guideline criteria (5) to get reliable numbers. Therefore, we defined the patient groups as follows:

- CC: Chronic cough > 8 weeks.
- TRCC: “therapy resistant chronic cough“, all patients unresponsive to any treatment irrespective of underlaying disease, including RCC/UCC patients.
- RCC/UCC DGP group (refractory CC and unexplained CC): all patients with “therapy resistant CC” according to DGP guideline = unresponsive to treatment of the following underlaying diseases: asthma, non-asthmatic eosinophilic bronchitis (NAEB), upper airway cough syndrome (UACS), gastro-esophageal reflux disease (GERD) ACE-inhibitor cessation (ACI).

For TRCC patients, physicians also documented more detailed information about cough characteristics, diagnosis (multiple answers possible), treatment, and comorbidities. There were audits in 2 randomly selected sites to verify the accuracy of the data.

### Patient selection

Patients fulfilling all the following criteria were consecutively included in the detailed documentation for TRCC: ≥18 years; established/definitive specialist’s diagnosis of CC; written informed consent. Exclusion criteria were: CC that was responsive to the treatment of the underlying disease such as asthma, COPD etc.; active smoking (however, former smoker since >12 months and < 20 pack years were eligible); active participation in another study or clinical trial.

### Data management

Data were collected online using an electronic data capture system (solution “Ebogen”). The collected data were enhanced with further data sources to improve the planned estimation of the prevalence and to verify required assumptions of the estimate.

Data sources were:

- IQVIA asthma prescription data of 2020.
- The National Health and Wellness Survey, KANTAR (11).
- Population data from Federal Statistical Office.

A total of 16 active practices, and 21,244 overall patient observations form the data basis from VICHAS. Prescription data from IQVIA provided 958 pulmonary facilities with 2,251,932 (unique) patients for the data merge. This enhanced data forms the basis for a descriptive preliminary verification of the representativeness of the VICHAS sites and the data basis for the subsequent modeling.

### Statistical framework

For statistical reporting categorical variables were shown in frequency tables including information on absolute and relative cough frequencies as well as the number of missing values. Continuously distributed variables were analyzed by reporting the sample median and interquartile range.

All values are presented as they were reported by the sites, with the following exceptions: if the day (e.g., of medication, diagnosis) was unknown, it was imputed as day 16 of the month, if the month was unknown, it was imputed as month 7. Missing years were not imputed.

Statistical analyses were conducted with the software package SAS version 9.4 (SAS Institute Inc., Cary, NC, United States).

### Modeling of prevalence estimates

Due to the restrictions of the Covid-19 pandemic and the weak implemented definition of RCC/UCC, a conventional estimate of the prevalence of RCC and UCC among patients visiting a respiratory outpatient practice was not feasible with the present study data. The decision to adopt this model was taken on the grounds that it would provide a more accurate estimation of the probabilities if independent parameters were included. Therefore, generalized linear mixed models (GLMM) were used as statistical procedure, which assumes normal (Gaussian) random effects and data can have any distribution in the exponential family. This allows modeling of the relative proportions of patients in Germany, even though the collected data were incomplete due to Covid-19. On the basis of the enhanced data, estimated probabilities (dependent parameter) could be made in the course of modeling and at the same time independent parameters and gaps in the data documentation could be considered and bypassed. The model calculations were performed for each target parameter (diagnosis) separately and resulted in an estimate of the relative proportion of patients in pulmonologist practices. These relative proportions provided the basis for further steps and conversions (Tables Estimates 1–4). In total, the following steps were taken to estimate prevalences:

- Estimate 1: Estimated probability of a specific cough diagnosis among patients referred to pulmonologist practices in Germany (general linear mixed model GLMM; mixed/ independent parameters: center effects, quarter of the year, number of pulmonologists per center).
- Estimate 2: Extrapolation and conversion to absolute frequencies (based on GLMM results and as stored in IQVIA prescription data).*
- Estimate 3A: Extrapolation to absolute frequencies in the German population of adults (≥ 18 years of age; using data from KANTAR and from the Federal Statistical Office), based on the assumption that 64/147 (43.5%)** patients consult a pulmonologist for CC.
- Estimate 3B: Extrapolation to absolute frequencies in the German population of adults (≥ 18 years of age using data from KANTAR and from the Federal Statistical Office), based on the assumption that 106/739 patients (14.4%)** consult a pulmonologist for CC.
- Estimate 4: Conversion and summary (of Estimates 3A + 3B) to estimated prevalences, based on German population of adults (≥ 18 years of age; 69,411,087 Mio adults in Germany in 2020).
  * Conversion to absolute numbers were made using prescription data (IQVIA asthma prescriptions data for 2020 as a confirmatory analysis of the representativeness of the selected study sites). It was examined whether the VICHAS practices were statistically different from all pulmonary practices to prove the representativeness of VICHAS practices. Asthma was the main diagnosis in the VICHAS study (~60%). Therefore, prescription data for asthma medications from the IQVIA database were used to compare the usage of asthma medications in the VICHAS study vs. the use of asthma medications in all other pulmonologist practices. For this purpose, the number of patients and the number of pulmonologists were compared in the prescription data from IQVIA (enhanced by VICHAS data). It was found that the VICHAS practices are not statistically different from all pulmonary practices. Representativeness of VICHAS practices could be assumed.
  ** In the KANTAR survey, participants were asked about referral to a specialist to have their cough clarified. A pulmonological examination of CC without reference to time was carried out in 64 of 147 patients (Estimate A). 106 of 739 patients with a CC visited a pulmonologist in the last 12 months (Estimate B).

## Results

**Figure 1:**
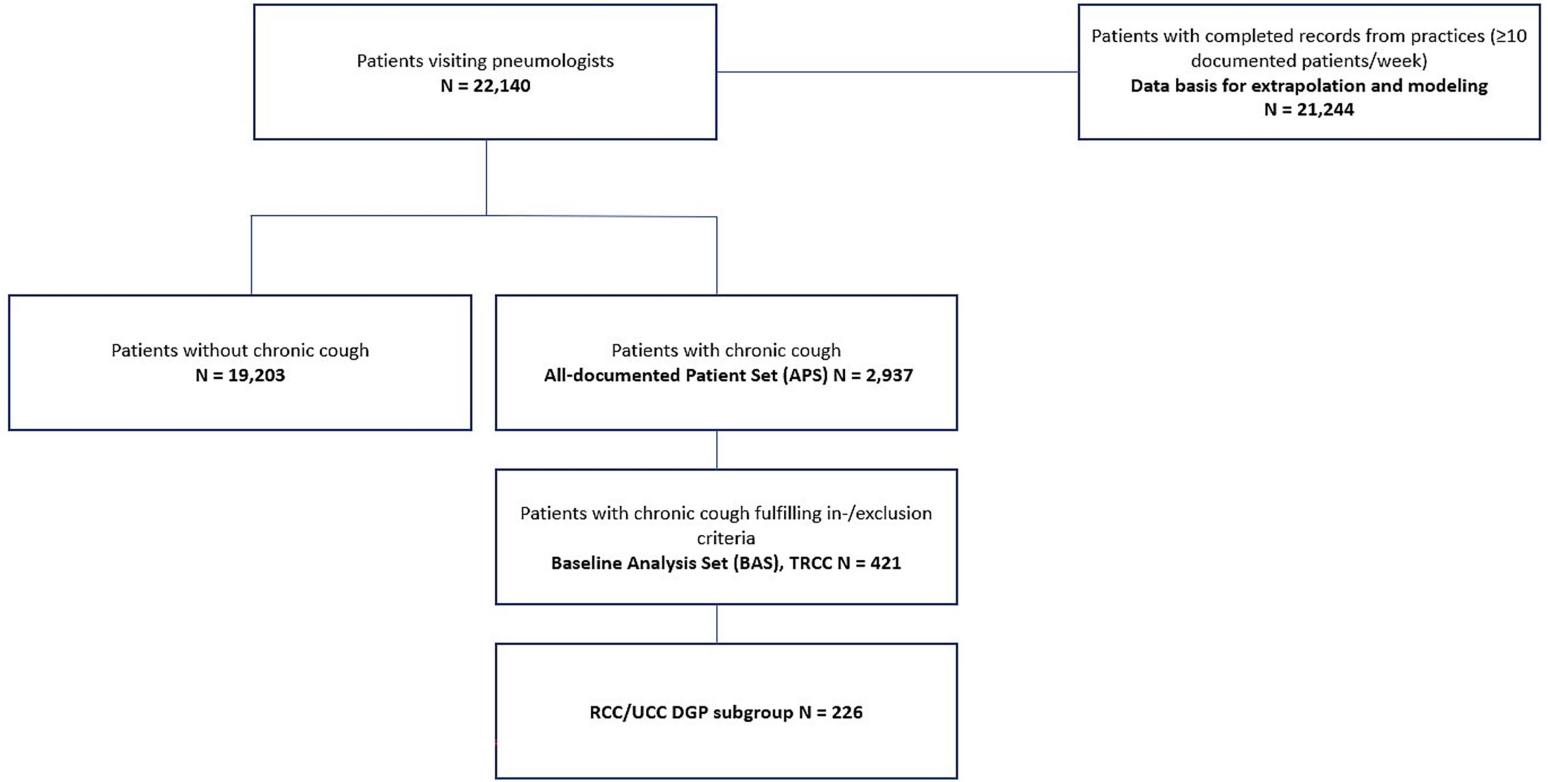
Patient disposition. The All-documented Patient Set (APS) consists of all patients with chronic cough documented by the participating sites (*n* = 2,937). The Baseline Analysis Set (BAS) consists of all patients of the APS with established specialist’s diagnosis of TRCC, RCC or UCC and signed written informed consent for the patient study and fulfilled the inclusion and exclusion criteria (*n* = 421). For quality assurance purposes, a number of weekly data were rejected and data were not used for prevalence calculations. This results in the Extrapolation Analysis Set (EAS), which consists of all data with complete documentation and weekly data of sites with at least 10 patients/week (*n* = 21,244).

The study flow chart is presented in Figure 1. Pulmonologists documented a total of 22,140 patients. Among these, 2,937 patients had chronic cough (CC) in VICHAS, forming the All-documented Patient Set (APS). Of these, 421 (14.3% CC patients) had a confirmed specialist’s diagnosis of TRCC, constituting the Baseline Analysis Set (BAS). Additionally, 226 patients (7.7% CC patients) met the criteria for the RCC/UCC DGP group at the specialists’ level.

The numbers of patients in VICHAS did not change significantly due to the season (despite triggers such as cold air in the wintertime) or the type of respiratory practice (e.g., solo practices, group practices, employed physician practices).

### Characteristics of patients

The mean age of TRCC patients was 59.7 ± 15.4 years, with 71.3% being female. Within this TRCC population, which included patients with conditions such as COPD and ILD, RCC was diagnosed in 77.9% (328/421) of the patients, and UCC in 22.1% (93/421). The primary causes of TRCC were controlled asthma (59.1%), and gastroesophageal reflux disease (19.1%; Table 1).

**Table 1:** Suspected causes for chronic cough.

Therapy resistant chronic cough (TRCC) cause	N	%
Controlled asthma, but cough symptoms	192	59.1
Gastroesophageal reflux disease (GERD)	62	19.1
COPD	39	12.0
Sleep apnoea	25	7.7
Chronic rhinosinusitis	21	6.5
Pulmonary fibrosis	17	5.2
Bronchiectasis	13	4.0
Caused by medication	8	2.5
Left heart failure and pulmonary congestion	2	0.6
Other causes		
Single	52	16.0
Single or combined*	111	34.2

Sample *n* = 325 from the Baseline Analysis Set (*n* = 421). Of the 421 TRCC patients, 93 had UCC and therefore were excluded from this table. Multiple answers possible, therefore 100% are exceeded. *Combined with other causes named in the list. For prevalence estimation modeling only the diagnoses of RCC and UCC according to the DGP guideline were used.

A total of 226 patients met the RCC/UCC DGP group definition criteria for inclusion in the prevalence estimation. The most frequent cough-related complications or comorbidities reported were: sleep disturbances in 16.3%, depression in 6.3%, and incontinence in 5.5% of the female patients. Other conditions were reported at a frequency of 0.5% or less. Notably, there were no reports of cough related rib fractures.

### Pathway of diagnosing CC

Among TRCC patients, 61.9% had a previous diagnosis of CC, provided by their general practitioner (*n* = 56; 21.8%), pulmonologist (*n* = 175; 68.1%) or another specialist (*n* = 26; 10.1%) (multiple answers were possible). On average, the CC diagnosis in TRCC patients was made 6.4 ± 7.6 years (median: 3.0 years) before the study.

Most TRCC patients (96.0%) had consulted one or more specialists for their condition before entry into the study. Specifically, 297 patients (73.5%) had consulted a pulmonologist, 103 patients (25.5%) an ear nose and throat specialist, 56 patients (13.9%) a gastroenterologist, and 128 patients (31.7%) any other specialist. Regarding the number of specialists consulted, 259 patients (64.1%) had seen one specialist, 126 patients (32.2%) 2–3 specialists, and 19 patients (4.7%) more than 3 specialists.

A wide variety of medical examinations were performed to confirm the diagnosis and exclude other conditions. Screening for cough-causing drugs was done in 40.6% of the patients including discontinuation of the identified drugs. Diagnostic procedures included chest X-rays in 75.2% of the patients, CT scan of thoracic organs in 24.6%, pulmonary function tests in 98.1%, test for unspecific bronchial hyperreactivity in 42.5%, measurement of fractional nitric oxide concentration in exhaled breath (FeNO) in 29.1% (not reimbursed in Germany), and ear nose throat (ENT) examination in 28.6% of the patients (details in Table 2).

**Table 2:** Medical examinations in chronic cough.

Medical examinations	*n*	%
Screening for underlaying medical conditions	419 (of 421)	99.5
Screening for drugs causing cough	170	40.6
Discontinuation of cough causing drugs	24	5.7
Chest X-ray	315	75.2
CT of thoracic organs	103	24.6
Pulmonary function test	411	98.1
Test for unspecific bronchial hypersensitivity	178	42.5
Bronchoscopy	35	8.4
Neurological examination	7	1.7
Eear Nose Throat examination	120	28.6
FeNO test	122	29.1
Cardiac examination	67	16.0
Reflux diagnostics	53	12.6
Gastroscopy	50 (of 52)	96.2
Impedance pH measurement	6 (of 52)	11.5
Oesophagogastroduodenoscopy	4 (of 52)	7.7
Oesophageal manometry	4 (of 52)	7.7
Oesography	1 (of 52)	1.9

Sample from the Baseline Analysis Set, based on *n* = 419 respondents if not indicated otherwise. Multiple answers possible, therefore 100% are exceeded.

### ICD-10 categories

The most common ICD-10 diagnosis for the current visit was R05 Cough (79.8%), followed by J40-J47 Chronic lower respiratory diseases (57.5%), K21.9 Gastro-oesophageal reflux disease without oesophagitis (12.8%), J30-J39 Other diseases of the upper respiratory tract (9.5%), and G47.3 Sleep apnoea (6.7%).

### Medications and other therapeutic measures

A majority of TRCC patients reported using medication for RCC (56.4%) and UCC (72.0%) in the past. Current medications included inhaled corticosteroids (ICS) in 26.8%, ICS/LABA combinations in 45.6%, herbal antitussives in 23.3% and over-the-counter cough syrup in 9.0%. Codeine was used by 4.3%, morphine by 0.7%, and gabapentin by 0.2%. Detailed information is shown in Table 3. Non-drug therapeutic measures were infrequently reported, with respiratory physiotherapy in 8.2% previously and in 1.7% currently and logopaedics/speech therapy used in 3.8% previously and in 0.2% currently. According to the patients’ self-rating of the efficacy, only 38.1% reported partial improvement of CC with their medication.

**Table 3:** Medication (past and current) for treatment.

Medication	In the past *n*	%	Currently *n*	%
Morphine sulfate	6	1.4	3	0.7
Codeine	85	20.2	18	4.3
Noscapine	19	4.5	4	1.0
Dextromethorphan	5	1.2	1	0.2
Dihydrocodeine	2	0.5	1	0.2
Levodropropizine	0	0.0	1	0.2
				
Benproperine	2	0.5	1	0.2
Herbal antitussives	140	33.3	98	23.3
Acetylcysteine	48	11.4	11	2.6
Carbocysteine	2	0.5	0	0.0
Ambroxol	51	12.1	5	1.2
Bromhexine	6	1.4	0	0.0
				
Sodium chloride solution (inhalation)	49	11.6	25	5.9
Herbal expectorants	14	3.3	8	1.9
Cough syrup (over-the-counter)	78	18.5	38	9.0
Inhaled corticosteroids (ICS)	91	21.6	113	26.8
ICS/LABA *	121	28.8	192	45.6
Inhalative anticholinesterase drug	44	10.5	57	13.5
Proton pump inhibitor (PPI)	38	9.0	47	11.2
Gabapentin	1	0.2	1	0.2
Pregabalin	0	0.0	0	0.0
Amitriptyline	1	0.2	0	0.0

Sample from the baseline analysis set (*n* = 421). Multiple answers possible, therefore 100% are exceeded. *Includes ICS/formoterol.

### Estimated prevalence

According to the model described and the inclusion of the RCC/UCC DGP group in the calculation, some of the confidence intervals, especially for the RCC/UCC group, are wide or undefined, indicating limitations in the precision of these prevalence estimates. The following prevalence values were estimated for the adult German population (Tables Estimates 1–3 with intermediate and Estimate 4 final results):

- CC: 0.83 (confidence interval 0.29;1.41) and 2.32 (confidence interval 0.69;5.48).
- RCC/UCC DGP group: 0.21% (confidence interval: N/A; 0.42%) and 0.64% (CI: N/A; 1.28%).

## Discussion

This cross-sectional study, conducted shortly after the publication of the new definitions for RCC and UCC, is the first of its kind in Germany. It provides specific information about the prevalence and characteristics of patients with CC, particularly those with treatment resistant chronic cough, including RCC and UCC. While recent data on CC have been reported for several countries, including Germany from a large patient panel named 2020 EU4 NHWS (KANTAR) (The National Health and Wellness Survey by KANTAR) (11), there were no data from secondary care office-based physicians. Such data are of particular interest because patients with long lasting CC are often referred from the primary care physician to pulmonary specialists for a detailed diagnostic work-up. Moreover, Germany lacks dedicated cough specialists and multidisciplinary cough clinics. This is also why patients consult different doctors over long periods of time without receiving a satisfactory diagnosis or therapy (10).

In contrast to previous studies including the KANTAR survey, our current study aimed to differentiate between RCC and UCC, revealing that RCC was much more frequent compared to UCC (77.9% vs. 22.1%). Chronic respiratory diseases are commonly associated with cough. This can obscure the distinctions between CC as a symptom of conditions such as interstitial lung disease and CC triggered by upper airway cough syndrome (UACS), cough variant asthma, or gastroesophageal reflux disease. The DGP and ERS guidelines explicitly name conditions like asthma (cough as asthma equivalent), non-asthmatic eosinophilic bronchitis, upper airway cough syndrome including rhinosinusitis and pharyngolaryngitis, gastro-oesophageal reflux disease (GERD) and ACE inhibitor use as potential triggers for RCC. Our study found that most patients with RCC had asthma (59.1%), GERD (19.1%), and chronic rhinosinusitis (6.5%). It is crucial that the underlaying medical condition that is suspected to cause CC is treated adequately according to the corresponding guideline. However, if the cough does not respond, the RCC definition is met.

Based on data from IQVIA, KANTAR and the Federal Statistical Office, extrapolations resulted in prevalence estimates in adults for CC, depending on the model used, of 0.83 and 2.32%. For RCC/UCC according to DGP guideline definition (RCC/UCC DGP group), the estimates were 0.2 and 0.6%. For comparison, in the German cohort of KANTAR, the patient self-reported 12-months prevalence of CC was 4.9%. The definition of RCC/UCC in VICHAS was determined by pulmonologists.

However, the results of the VICHAS study need to be interpreted with caution, as the estimates - especially for UCC - are probably biased due to the small number of UCC patients in the study and the fact that RCC/UCC diagnoses were just introduced at the time of the study. If the confidence intervals are considered, the distribution when using the two assumptions for chronic cough is between a minimum of 0.29 and a maximum of 5.48. For CC according to the DGP definition (RCC/UCC DGP group), the distribution is between a minimum of “not applicable” and a maximum of 1.28. The maximum CI for CC patients in Germany of 5.48% in VICHAS aligns with KANTAR data (4.9%). Due to various limitations, especially Covid-19, it could be assumed that VICHAS underestimates the prevalence of CC and RCC/UCC on average. Based on the data from KANTAR, the prevalence could be more aligned with the upper limit of the confidence interval, suggesting a prevalence of 5.5% for CC in the adult German population and 1.3% for RCC/UCC.

The mean age of patients with TRCC in the present study was 59.7 years, which was higher than in the population-based Austrian LEAD study (53.8 years) (18), and very similar to the COUGH-1 and COUGH-2 gefapixant randomized controlled trials (59.0 years) (19). The age distribution aligns with the ERS guidelines, which describe a peak between 50 and 60 years of age (3). Additionally, the preponderance of females described in the guidelines was present in VICHAS (71.3%), similar to LEAD (74.2%) and the gefapixant trials (COUGH-1 74.2%, COUGH-2 74.9%). It is well documented that the cough reflex is more sensitive in women compared to men (20).

Patients in VICHAS had a long history of cough, with CC lasting an average of 6.4 years. This is consistent with the LEAD study, where the median duration was 3 years, with 32% of patients experiencing symptoms for longer than 5 years, and the COUGH trials, which reported a mean of 11.6 and 11.2 years (18, 19).

It was striking that, according to all guidelines, the great majority of the patients underwent imaging and almost all patients had a lung function test. A FeNO test was carried out less frequently, likely because this test is an out-of-pocket expense for patients. The diagnostic algorithm from the guidelines appears to be followed in clinical practice, indicating that patients are diagnosed thoroughly and that possible causes for cough are investigated or excluded, respectively.

High rates of underlying medical conditions are a typical finding in cough studies (12). For example, in the recently published German arm of KANTAR study, individuals with CC in general (not limited to RCC/UCC) had additional diagnoses of the respiratory system in 71% of the cases, the digestive tract in 34%, and cardiovascular diseases in 28%. Consequently, they had higher morbidity scores compared to patients without CC (11).

Most studies have collected CC data, i.e., cough lasting >8 weeks without differentiating whether it was a symptom or RCC/UCC. Given the wide range of possible underlaying diseases, patients could also have presented with lung cancer or tuberculosis. However, differentiating these individual, well defined, and treatable groups of a CC is crucial for targeted treatment.

When considering the comorbidities, the type and pattern were consistent with those reported in the DGP and DEGAM guidelines. The rate of self-reported depression (6%) in our study was the same as in studies conducted in China (21) or in an urban community in South Korea (22). Additionally, a recent analysis from the Rotterdam study indicated that individuals with CC have a substantially higher burden of depressive symptoms, independent of commonly associated risk factors and other comorbidities such as smoking and asthma (23).

However, the rate of incontinence (6%) in our study was much lower than the one reported from a specialized cough center in the United States, where 63.3% of women reported stress incontinence using a targeted questionnaire (24). This discrepancy can likely be explained by under-reporting within our study. Incontinence, in particular, should be inquired more actively, as patients are often reluctant and ashamed to report this condition (25).

In Germany, codeine and noscapine are approved for short-term treatment of acute cough. However, at the time of the study, no medication was approved for the long-term treatment of CC, particularly RCC and UCC. In our study of RCC/UCC managed by pulmonologists, a broad variety of drugs were used, including off-label medications. Inhaled corticosteroids were among the most frequently used therapies, likely reflecting the assumption that the cough was due to cough variant asthma or eosinophilic bronchitis – conditions for which this treatment is recommended in both DGP and ERS guidelines as a trial for up to 4 weeks. Herbal antitussives were also used frequently. Notably, codeine, which was among the most frequently used drugs prior to the documented visit, played a minor role among the current medications.

It was not documented, however, whether medications such as ICS/LABA combinations or herbal antitussives were prescribed specifically for the treatment of an underlying disease (e.g., asthma or GERD) or for the management of chronic cough itself. However, based on the inclusion criteria and clinical context, it is more accurate to assume that patients were treated for chronic cough, potentially caused by these underlying conditions, rather than being treated directly for asthma or GERD per se. Thus, most of the observed therapies likely reflect attempts to manage cough symptoms—consistent with guideline recommendations to treat the suspected cause of cough empirically, even in the absence of a definitive diagnosis. This distinction is important for interpreting treatment patterns and highlights the clinical uncertainty that often accompanies RCC and UCC. Off-label neuromodulators such as gabapentin, pregabalin, and amitriptyline—although guideline-recommended—were rarely prescribed. This, together with the variability in treatment approaches and the fact that only 38% of patients reported partial symptom relief, underscores the limited effectiveness of available therapies at the time. Similar levels of burden and low treatment satisfaction have also been reported in other real-world populations with chronic cough (26). These findings highlight a significant unmet clinical need. Emerging therapies such as P2X3 receptor antagonists were not yet available during the study period but may offer future benefit.

## Limitations

At the time of study planning, the RCC population was not explicitly defined according to the DGP criteria in the study protocol. The definitions of RCC and UCC were introduced in 2019 by the German DGP guideline and not fully implemented at the time of the study. The participating sites had considerable interest and knowledge about the topic and the definitions used for CC, RCC and UCC. Nevertheless, other treatment resistant CC patients with conditions such as COPD or ILD were included into the study.

The VICHAS study began in January 2020 and was conducted in pulmonary practices. Due to the Covid-19 pandemic, the evaluation had to be paused from April until end of June because the focus of the participating sites had shifted accordingly. Additionally, patients with CC may have avoided coming to the practices in 2020 due to their condition and the stigmatization of these patients, which may have lowered prevalence estimates.

At the time of the study, RCC and UCC had not yet been recognized as distinct diagnostic entities in Germany. A specific German ICD-10 code was introduced only 3 years later, and an international ICD-10 code is still lacking. This likely contributed to diagnostic under-recognition and uncertainty in the resulting prevalence estimates. For estimating the prevalence data, an analysis of the RCC/UCC population according to DGP criteria (RCC/UCC DGP group) was performed in addition to the TRCC population, which included all refractory cough cases. The small number of sites and patients due to the Covid-19 pandemic may have influenced statistical modeling. Modeling is always accompanied by simplifications and uncertainties.

Several other methodological considerations must be acknowledged when interpreting the findings of this study. As a cross-sectional investigation, it captures only a snapshot in time and therefore cannot provide insights into the temporal evolution of CC, including disease progression, variability in symptom burden, or treatment responsiveness over time. These clinically important aspects can only be addressed through prospective longitudinal studies.

We did not perform subgroup analyses by age, sex, or comorbidities due to limited subgroup sizes, which may mask meaningful heterogeneity. A validated cough-specific quality-of-life instrument such as the LCQ or CQLQ was not used, which limits the assessment of patient-reported outcomes. The study was designed to investigate CC, RCC and UCC under clinical practice conditions in pulmonologists’ offices, and results may not readily be extrapolatable to other settings. As opposed to other disease entities such as asthma or COPD, pulmonologists do not undergo special training or specialization in cough. No dedicated “cough clinics” exist in Germany. Presence of selection bias toward more severe patients who were transferred to specialists in pneumology must be assumed. Reporting bias could not be excluded concerning the absence of the possibility to validate patients´ data entries might have caused the low number of comorbidities.

## Conclusion

This is the first study in a secondary care setting estimating the prevalence of RCC and UCC according to the DGP definition in outpatients in Germany. A model projected the prevalence of RCC/UCC patients to the German population based on two assumptions (A and B) of the proportion of CC patients presenting at pulmonology clinics as 0.21% (Estimate A: confidence interval: N/A; 0.42%) and 0.64% (Estimate B: CI: N/A 1.28%), respectively.

The present study provides estimates of the prevalence of patients with CC and RCC/UCC DGP group in the German adult population, seen by office-based pulmonologists, who had not yet fully applied the new definitions for RCC/UCC of the DGP guidelines at the time of the survey. The findings are in line with those from the German cohort of KANTAR that used a patient-focused methodology as well as other recent studies. Diagnostic procedures indicate that often lung specialists participate in evaluation. In terms of therapy, in the absence of approved drugs for RCC/UCC, many patients receive medications off-label at least temporarily for their condition, alongside with treatments of possible underlying diseases. Patients have a long history of suffering, as CC lasts for an average of 6 years. The data support the importance of RCC and UCC in the clinical setting, and the need to improve therapy and to develop new therapeutics.

## Data Availability

The raw data supporting the conclusions of this article will be made available by the authors, without undue reservation.
